# Are Populist Leaders Creating the Conditions for the Spread of COVID-19? Comment on "A Scoping Review of Populist Radical Right Parties’ Influence on Welfare Policy and its Implications for Population Health in Europe"

**DOI:** 10.34172/ijhpm.2020.124

**Published:** 2020-07-14

**Authors:** Martin McKee, Alexi Gugushvili, Jonathan Koltai, David Stuckler

**Affiliations:** ^1^London School of Hygiene and Tropical Medicine, London, UK.; ^2^Department of Sociology and Human Geography, University of Oslo, Oslo, Norway.; ^3^Carlo F. Dondena Centre for Research on Social Dynamics and Public Policy, Bocconi University, Milan, Italy.

**Keywords:** Populism, Political Determinants of Health, COVID-19

## Abstract

Do populist leaders contribute to the spread of coronavirus disease 2019 (COVID-19)? While all governments have struggled to respond to the pandemic, it is now becoming clear that some political leaders have performed much better than others. Among the worst performing are those that have risen to power on populist agendas, such as in the United States, Brazil, Russia, India, and the United Kingdom. Populist leaders have tended to: blame "others" for the pandemic, such as immigrants and the Chinese government; deny evidence and show contempt for institutions that generate it; and portray themselves as the voice of the common people against an out-of-touch ‘elite.’ In our short commentary, focusing on those countries with the most cases, we find that populist leaders appear to be undermining an effective response to COVID-19. Perversely, they may also gain politically from doing so, as historically populist leaders benefit from suffering and ill health. Clearly more research is needed on the curious correlation of populism and public health. Notwithstanding gaps in the evidence, health professionals have a duty to speak out against these practices to prevent avoidable loss of life.

## Introduction

In the accompanying paper, Rinaldi and Bekker review evidence that the ideology pursued by populist radical right parties in Europe is harmful for health.^1^ Their analysis builds on a small but growing body of literature on what has been described as the political determinants of health.^2,3^ The decisions that politicians make shapes the conditions in which people live and the choices, many with consequences for the health, that are available to them. This is crucial during disease outbreaks, like the coronavirus disease 2019 (COVID-19) pandemic, which requires politicians to set aside ideology and act on a rapidly emerging and uncertain body of public-health evidence.

 Here we extend the important work of Rinaldi and Bekker to ask, are populist leaders creating conditions which contribute to the spread of COVID-19?

Before doing so, we first note that the COVID-19 pandemic is unique in recent times in generating broadly comparable data (albeit far from perfect) in every country in the world with a delay of days or weeks at most. Historically, publication of health data, if it takes place at all, is often long delayed and, in many cases, lacking the granularity necessary to provide a detailed assessment of how countries are performing in improving the health of their populations. This means that the health consequences of policies only become apparent long after the decisions that gave rise to them. Now, measures of health are leading the news. Heads of government in many countries are appearing, in some cases daily, to share the news on the progress of the pandemic with their people. Anyone can go to the Johns Hopkins website to track, in almost real time, numbers of cases and deaths.^4^ A leading global newspaper, the *Financial Times*, has become a key source of information on comparative COVID-19 data, discussing their strengths and weaknesses.^5^ These data make it possible for politicians to be held to account for the ability to protect the health of their populations in almost real time, in marked contrast to the situation described above when the consequences of their decisions for health are only seen years later.

 There appears to be a striking correlation between countries led by politicians who support populist messages and the poor performance in responding to COVID-19. As of June 25, 2020, the United States, Brazil, Russia, India, and the United Kingdom occupied, in that order, the top five positions ranked by numbers of COVID-19 cases in the Johns Hopkins dashboard. Together, they account for 51% of all cases worldwide but only 27% of the world’s population.

 There are four potential mechanisms for such a link between populism and COVID-19 spread. We take each in turn.

 Mechanism 1: Blaming Outsiders and Victims

Rinaldi and Bekker examine the populist radical right, with its combination of nativism (a policy of protecting the interests of persons born in a state against those of immigrants) and authoritarianism (a policy favouring strict obedience to authority over personal freedoms). This is logical given the authors’ focus on welfare policy and health. However, when seeking to understand responses to COVID-19 we believe that it is appropriate to look at populism even more broadly. In this respect, we additionally consider one mechanism linked to a definition of populism proposed by Albertazzi and McDonnell, who see it as an ideology that “pits a virtuous and homogeneous people against a set of elites and dangerous ‘others’ who are together depicted as depriving (or attempting to deprive) the sovereign people of their rights, values, prosperity, identity, and voice.”^6^ This definition brings together populist politicians on the left, such as Venezuela’s Nicolás Maduro, with those on the right, such as Brazil’s Jair Bolsanaro, as well as those whose politics are influenced heavily by nationalism or religion, such as Turkey’s Recep Tayyip Erdoğan or India’s Narendra Modi. Others adopting populist approaches include America’s Donald Trump, Russia’s Vladimir Putin, and the UK’s Boris Johnson.

The populist approach that these politicians have adopted has involved appealing to groups in society that have been left behind,^2^ for various reasons including the consequences of loss of traditional industries and who have struggled to adapt to changing circumstances, often because of a lack of the necessary skills. Understandably, these groups have sought someone to blame for the misfortune. Populist leaders have seized upon their disaffection, developing a narrative in which their misfortunes are due to the actions of others. These are often those who are identifiably different, for example because of their dress or the colour of their skin. Thus, Donald Trump explicitly invoked COVID-19 as a justification for increasing restrictions on workers coming from abroad.^7^ The populist narrative continues by developing a worldview in which the circumstances that have allowed people to be left behind have been created by a remote and unfeeling elite. Into this situation, the strong leader emerges as the saviour of his (and almost always it is his rather than her) people.

This ‘insider-outsider’ narrative has served populist politicians well, attracting the votes of those who feel they have been left behind.^8,9^ The populist politician offers hope of a better future. It matters little that they often fail to deliver. Any failure is always someone else’s fault, and particularly those from the “other” group or the elite that protects them.

 The difficulty arises when the threat is not from the “other” group but, as with COVID-19, it is from a microorganism. Just as, to employ a widely used cliché, viruses pay no attention to national borders, nor do they have passports or citizenship. Of course, this did not prevent President Trump from blaming the Chinese, or ‘Kung Flu’ virus, or Hindu nationalists from blaming Muslims.

 Mechanism 2: Contempt for Institutions

 When the history of the COVID-19 pandemic is written, it will be possible to identify many individual decisions by politicians that influenced its course. Among them, and likely the most important, will be the timing of the decision to impose restrictions on movement. Others will include choices about how and when to implement testing and tracing strategies, how messages were communicated, and how the material necessary to respond to the pandemic was procured. However, underlying all of these we can identify two characteristics of populism that have contributed to the situation in which all of these countries have done especially badly during the pandemic.

The first is a contempt for the traditional institutions that are populated by the elites, sometimes described as “enemies of the people.”^10^ Populist leaders are reluctant to be bound by institutional constraints, such as constitutions and courts. By switching between being president and prime minister, Vladimir Putin has managed to circumvent the constitutional restriction on terms of office. Boris Johnson was found to have prorogued Parliament illegally. Jair Bolsonaro has spoken of his desire to abolish the Supreme Court. It is, however, their approach to public health institutions that are particularly important here. These are characterised by a combination of neglect, denying necessary funding or leaving key positions unfilled, or hostility. Thus, three months before the emergence of COVID-19, the Trump administration closed down the USAID funded PREDICT programme, developed to provide early warning of possible pandemics.^11^ In the UK the cabinet’s Threats, Hazards, Resilience and Contingency Committee was axed by Boris Johnson a few days after assuming office.^12^ Jair Bolsanaro fired two health ministers within weeks.^13^ In 2017 Narendra Modi cut the planned budget of the National Health Mission, India’s public health service, by 20%.^14^ Donald Trump attempted to impose similar cuts on the Centers for Disease Control but was blocked by Congress.^15^ The organisation did, however, have to reduce substantially its outpost in Beijing, established to provide information on emerging viruses. The situation in Turkey was different. There, the health system was weakened by Recep Tayyip Erdoğan’s dismissal of tens of thousands of civil servants, including leading virologists, following an attempted coup.^16^ In Venezuela the loss of institutional capacity was a result of economic collapse due to mismanagement.^17^

 Mechanisms 3: Denialism

The third, and related issue is rejection of evidence. Just as they are reluctant to be constrained by institutions and their rules, so they reject the laws of science, discovered and promoted by the elites, and their consequences. Rather, they adopted the tactics associated with denialism, including promotion of conspiracy theories, cherry picking evidence, citing false experts, moving goalposts, and employing a range of logical fallacies.^18^

Even though the rapid growth of the pandemic was apparent from early on, populist politicians were in denial about its potential effect on their countries. In early March 2020, Boris Johnson advised the public that “we should all basically just go about our normal daily lives’’ providing they washed their hands frequently, and boasted of shaking hands with infected patients.^19^ Later in March, Jair Bolsanaro described the pandemic, which by then had accounted for less than 20000 deaths worldwide, as a “media trick.”^20^ Many populist politicians have promoted treatments lacking any evidential basis, most notably Hydroxychloroquine, in a possibly unique example of agreement Donald Trump and Nicolás Maduro.^21^

While there is a wealth of evidence that consideration of all the evidence leads to better decisions, populist politicians reject anything that challenges their beliefs. Hence, when the director of the US health department’s Biomedical Advanced Research and Development Authority challenged Trump’s support for hydroxychloroquine he was fired.^22^ Of course, faced with a situation they cannot control, it is understandable that populist politicians, used to spreading positive messages, will clutch at straws. Maduro had previously advocated drinking tea made from lemongrass and elderberry tea.^21^ However, in Trump’s case, it has become impossible to ascertain whether his increasingly bizarre statements reflect a deliberate attempt to create hope of a cure or whether they simply reflect his profound ignorance of basic science, as when he commented that the coronavirus “has gotten so brilliant that the antibiotic cannot keep up with it.”^23^

The prominent cases of populist leaders mismanaging the crisis described above is consistent with a recent quantitative study on political leaders’ responses to the COVID-19 pandemic in 94 countries from around the world.^24^ The results of this analysis suggest that governments headed by populist leaders delivered a weaker response to COVID-19 than non-populist ones by implementing fewer closures and less robust health countermeasures during the onset of the crisis. Thus it would seem that the consequences of apparently idiosyncratic circumstances associated with populism for the spread of COVID-19 in three of the worst affected countries, the United States, the United Kingdom, and Brazil, are representative of the broader developments in the world, especially considering that in that multi-country study the results are adjusted for countries’ economic capacity, demographic composition, earlier experience with pandemics such as severe acute respiratory syndrome (SARS), the level of democracy, and time elapsed since discovery of a country’s first confirmed COVID-19 case.

 Mechanism 4: Suspicion of Elites

While perhaps less direct than the key mechanisms highlighted above, it is also important to highlight the intersections between populist rhetoric, the media, and COVID-19. Populist politicians commonly position the media—particularly outlets that criticize their policies or messaging—as members of the ‘corrupt elite,’^25^ or the “enemy of the people,” in the case of Donald Trump. Yet, during a pandemic, experts must rely heavily on mainstream media platforms to generate public health awareness and articulate the steps that must be taken to mitigate risk.

This dynamic creates an insidious pathway through which populism may facilitate the spread of COVID-19. One recent study found that consumption of Fox News, a decidedly pro-Trump network, reduced propensity to comply with physical distancing measures across Zip codes in the United States.^26^ Another study found that obtaining news from mainstream broadcast media, such as NBC News and the *New York Times*, correlated with possessing accurate information about the disease’s lethality, and accurate beliefs about protection from infection. The study also found an association between watching Fox News and support for conspiracy theories, such as believing that the CDC is exaggerating the seriousness of COVID-19 to undermine the presidency of Donald Trump.^27^

 Reverse Causality: Suffering and Ill Health From COVID-19 Increasing Support for Populism

So far, we have considered the impact of populist politics on COVID-19, describing how it has inhibited the development of effective responses to the pandemic. But what about the impact of COVID-19 on populism? Will those who once supported these populist politicians turn against them as they see their friends and families die prematurely? The United States offers an opportunity to find out. Between March and the latest data, as of writing this paper, there was a sustained fall in cases in states and counties that voted for Clinton in 2016 while the reverse happened in those that had voted for Trump.^28^ Trump’s poll ratings have been falling but he still retains considerable support among his core vote. This may be explained, at least so far, by the cognitive biases that shape how people interpret news, itself related to the news channels they view. Thus, in late February 2020, early in the pandemic, 40% of Republicans were “not concerned at all” about a coronavirus outbreak in their communities, falling to 25% in mid March. In contrast, among Democrats the figure started at just over 5%, falling even further over the same period.^29^ This gap was corroborated by other data, including evidence that those living in Democrat supporting areas were more likely to search online for hand sanitizers.

COVID-19 may also shape the underlying forces that precede and correlate with populist sentiments and support. First, in addition to the devasting effects on population health, the pandemic has also precipitated an economic crisis. One recent well-publicized study found crisis-driven unemployment to be a substantial driver of political distrust and the subsequent rise in populist electoral support across Europe.^30^ Second, COVID-19 is intensifying the spread of online disinformation,^27,31^ which may further erode trust in experts and mainstream institutions, fuelling support for anti-establishment and populist narratives. Third, consistent with historical linkages between epidemic threats and xenophobia,^32^ emerging evidence suggests that the COVID-19 pandemic has triggered anti-Asian and anti-immigrant sentiments,^33,34^ views that align with populist rhetoric promising to protect a pure and (ethnically) homogenous people.

Although we will not know the actual implications of the pandemic for populist political parties and their leaders for some time, until votes in the next rounds of elections are counted, and these ramifications will be likely shaped by the human toll and the length of the pandemic, past experience suggest that some of the most memorable electoral upsets such as the 1930s’ rise of the Nazi Party in Germany,^35^ the election of Trump in the United States,^8^ and the Brexit referendum in the United Kingdom,^9^ were preceded by deteriorating population health. It is also clear that in the European countries in which populist parties until now played on a marginal role, radical right-wing groups are attempting to reap the benefits from COVID-19 by blaming, for instance, immigrants for spreading the virus in care homes in Sweden,^36^ smearing the idea of European solidarity in Italy,^37^ or in France promoting the narrative that Chinese scientists deliberately bred the virus in laboratory.^38^

 In sum, we are likely seeing the earliest evidence of a worrisome cycle: populism fuelling the spread of COVID-19, and, in turn, COVID-19 stoking the fires of populism.

Despite better public health data being available, more rapidly than ever before, in a climate where lies and disinformation are the new norm,^39^ we will need to develop strategies that can hold politicians to account for the health of the public. In democracies, this is achieved through the democratic process, but even in these countries this depends on voters being able to obtain fair and accurate information, which can be difficult where powerful vested interests dominate the mass media and if disinformation is disseminated widely on social media. The problem is much greater in non- or quasi-democratic states, where there are no free and fair elections and where those who speak out risk their careers or even their lives. In all of these circumstances, health professionals have a role to play, although it will vary according to circumstances. It places a great responsibility on those who can speak out, especially those in universities, who can be both advocates themselves and ensure that advocacy for health is included in undergraduate and postgraduate curricula.^40,41^ When they do, they will follow in a long and distinguished tradition.^42^

## Ethical issues

 Not applicable.

## Competing interests

 DS was supported by the European Research Council (ERC) ERC Grant 313590-HRES (D.S.) and Wellcome Trust (D.S.). Both the European Research Council and the Wellcome Trust had no role in the writing the manuscript, or the decision to submit the paper for publication.

## Authors’ contributions

 MM wrote the first draft, which all authors subsequently revised.

## Authors’ affiliations

^1^London School of Hygiene and Tropical Medicine, London, UK. ^2^Department of Sociology and Human Geography, University of Oslo, Oslo, Norway. ^3^Carlo F. Dondena Centre for Research on Social Dynamics and Public Policy, Bocconi University, Milan, Italy.
